# Dynamic-layer transformer-based reinforcement learning for observation-constrained multi-agent roundup scenarios

**DOI:** 10.1038/s41598-026-49608-7

**Published:** 2026-04-21

**Authors:** Xizhao Li, Ning Xu, Qingjia Chi, Hu Chen

**Affiliations:** 1 School of Information Engineering, Wuhan University of Technology, Wuhan, 430070 Hubei China; 2 School of Electrical Engineering and Automation, Anhui University, Hefei, 230601 Anhui China; 3 Wuhan Second Ship Design and Research Institute, Wuhan, 430064 Hubei China

**Keywords:** Multi-agent deep reinforcement learning, Dynamic transformer layers, Graph-based coordination, Observation constraints, Cooperative roundup, Engineering, Mathematics and computing

## Abstract

To address the challenge of cooperative roundup of maneuvering targets under limited perception, this paper proposes TransMARL, a transformer-based multi-agent reinforcement learning framework for observation-constrained coordination. The roundup task is formulated as a decentralized partially observable Markov decision process (Dec-POMDP), together with a local observation model and a dynamically updated interaction graph. The proposed framework combines a graph feature encoding module with a policy execution module to support decentralized decision-making under partial observability. A task-informed reward function is designed to encourage angular coverage, target approach, formation uniformity, and collision avoidance. In addition, the transformer depth is adaptively adjusted according to the team size as an empirically motivated design choice to balance representational capacity and computational cost. Experimental results in a 2D obstacle-free simulation environment show that, under the evaluated settings, TransMARL achieves competitive and often improved performance relative to the selected baselines, especially under constrained sensing radii. These results suggest that the proposed framework is a practical and scalable approach for cooperative control in observation-constrained multi-agent roundup scenarios, while its broader generalization and formal theoretical characterization remain to be further studied.

## Introduction

In recent years, multi-agent reinforcement learning (MARL) has gained increasing attention in domains such as collaborative control of unmanned systems, adversarial games, and distributed decision-making. In particular, dynamic tasks such as multi-target pursuit and evasion have become focal points of research. Traditional approaches, including differential game theory^14^ and heuristic control^28^, typically rely on precise modeling or hand-crafted rules, which are often inadequate for handling highly dynamic, uncertain environments^24^.

From a theoretical perspective, early work explored hierarchical reinforcement learning^33^ and evolutionary evaluation methods^23^, but these approaches often suffer from degraded policies and inefficient convergence in large-scale settings^5^. More recently, value decomposition methods and centralized training with decentralized execution (CTDE) frameworks have become widely adopted paradigms. For example, VDN^31^ introduces value decomposition to approximate the joint action-value function by summing individual value functions, enabling decentralized policy learning, while QMIX^25^ improves joint policy optimization via value function factorization. However, unlike methods that rely on expert trajectories, the present study adopts an end-to-end reinforcement learning formulation that learns cooperative strategies directly from partial observations, without requiring expert supervision.

Despite these advancements, MARL faces persistent challenges in complex dynamic environments. First, parallel policy updates induce a nonstationary environment, violating the i.i.d. assumption^9^. While CTDE frameworks partially mitigate this, conflicts persist between policy diversity and parameter sharing^13^.

Second, agents often require extended cooperation to achieve sparse terminal rewards, leading to slow learning and inefficient exploration. Techniques such as incremental goal exploration (IGE-MAPPO)^11^ enhance policy convergence by introducing auxiliary intermediate rewards to guide exploration towards long-term goals. Other methods leverage intrinsic motivation^30^to improve learning in environments with sparse feedback. However, these approaches often rely on carefully designed task decompositions and environment-specific shaping. Hare et al^12^. systematically evaluated the effectiveness of curiosity-driven strategies and auxiliary tasks in various sparse reward settings, but noted that exploration efficiency remains fundamentally limited when external reward signals are exceedingly scarce. To bridge the gap between dense and sparse reward scenarios, Luo et al^20^. proposed the Dense2Sparse framework, which combines the fast convergence of dense rewards with the robustness of sparse rewards, significantly improving performance and uncertainty tolerance in robotic manipulation tasks. Inspired by these insights, we design a task-guided reward function that integrates multiple factors–such as roundup angle, target distance, distribution uniformity, and collision avoidance–into a unified structure, with the aim of improving learning efficiency and coordination quality during training.

Third, in scenarios with limited communication or sensing, agents cannot perceive the global state. Graph neural network (GNN)-based methods^3,4,16,32^ attempt to model local interactions under these constraints but usually assume fixed topologies or homogeneous agents, limiting adaptability to dynamic and heterogeneous systems. Moreover, most MARL models require retraining or architectural modifications when the agent number changes. Although parameter sharing and modular networks^39^ offer partial solutions, scalability remains constrained in large-scale deployments.

Recent advances in Transformer-based architectures have expanded the design space of MARL. TIMAT^15^introduces a temporal information multi-agent Transformer that formulates MARL as a sequence modeling problem, capturing temporal dependencies and accommodating dynamic agent populations. Similarly, Micheli et al^21^. propose IRIS, a world model integrating discrete autoencoders with autoregressive Transformers, showing competitive sample efficiency by training policies through imagination without additional real-world interaction during policy learning. These studies suggest that Transformer-based architectures can offer potential benefits for generalization and scalability across varying agent numbers and observation settings.

In perception-constrained pursuit scenarios, Cheng et al^7^. design a perception-aware multi-agent pursuit framework that emphasizes decision-making under incomplete information, underscoring the importance of robust local observation encoding. Complementarily, the graph policy gradient (GPG)^17^ method leverages dynamic graph structures to model inter-agent dependencies effectively, particularly under local observability constraints. Dynamic topology-aware PPO (DTPPO)^36^ further extends this by dynamically adapting graph topologies during training, enhancing robustness to environmental variations. These studies mainly focus on interaction modeling and coordination mechanisms within MARL itself.

Beyond MARL-specific studies, recent work has highlighted the importance of robust representation learning, uncertainty awareness, and structured reasoning in intelligent decision systems. Robust latent representation under incomplete or perturbed observations has been explored through generative modality dropout and weakly augmented latent-variable learning^37,41^. Related efforts on efficient uncertainty quantification further suggest that explicitly modeling uncertainty can improve reliability under distribution shift and partial observability^6^. In addition, structured reasoning with reinforcement-learning-assisted information fusion has been shown to improve decision quality in complex inference settings^18^. Although these studies focus on different domains, they provide complementary perspectives on robustness, uncertainty handling, and representation stability that are conceptually relevant to cooperative decision-making under partial observability.

In this context, we propose TransMARL, a scalable MARL framework built upon a dynamic interaction graph and an adaptive-depth transformer encoder. The framework integrates a graph feature encoding module, which captures structured local interactions, with a size-invariant policy head that supports policy reuse across varying team sizes. Rather than claiming a universal advantage over existing graph-based MARL methods, our goal is to investigate whether an attention-based relational encoder with adaptive depth can provide an effective alternative for cooperative control under observation constraints.

In the experiments, we compare TransMARL with six representative baselines, including SAC^10^, GAT-MARL^32^, GPG^17^, MAPPO^40^, MADDPG^19^, and MATD3^1^. These comparisons are intended to provide a broad empirical reference across commonly used MARL paradigms, with particular emphasis on evaluating performance trends under local observation constraints and different team sizes. We therefore focus on whether the proposed design can maintain effective coordination and scalability in the studied roundup setting, rather than asserting universal superiority across all MARL architectures and information assumptions.

The main contributions of this work are summarized as follows:

- We propose an adaptive-depth transformer encoder for multi-agent interaction modeling, where the network depth is adjusted according to team size as a practical design rule to better match relational complexity under partial observability.
- We develop a graph-based local information fusion framework that combines dynamically constructed interaction graphs with multi-head attention to encode time-varying neighborhood relationships in observation-constrained settings.
- We design a size-invariant coordination architecture in which the policy and value representations remain compatible across different team sizes, enabling scalable training and cross-scale policy reuse within the evaluated task family.

The remainder of this paper is organized as follows. Section “Problem formulation” introduces the mathematical formulation of the multi-agent roundup task, along with the roundup success criteria. Section "Multi-agent roundup algorithm design" formulates the problem as a decentralized partially observable Markov decision process (Dec-POMDP)^22^ and details the design of the TransMARL algorithm, including observation encoding, dynamic graph construction, reward design, network structure, and training process. Section "Experimental design and results analysis" presents the experimental settings and analyzes the performance of TransMARL through ablation studies and scalability evaluations. Section "Conclusion and future directions" concludes the paper and discusses potential directions for future research.

## Problem formulation

The multi-agent roundup problem is widely applied in military roundup, intelligent security, and disaster rescue. The primary objective is to surround and capture an escaping target within a confined space using a team of cooperating agents. In this paper, we consider a finite, two-dimensional, obstacle-free environment, which involves the following elements:

- *N* homogeneous agents (pursuers) with local sensing and distributed decision-making;
- One dynamic escaping target governed by an artificial potential field (APF) strategy.

**Fig. 1:**
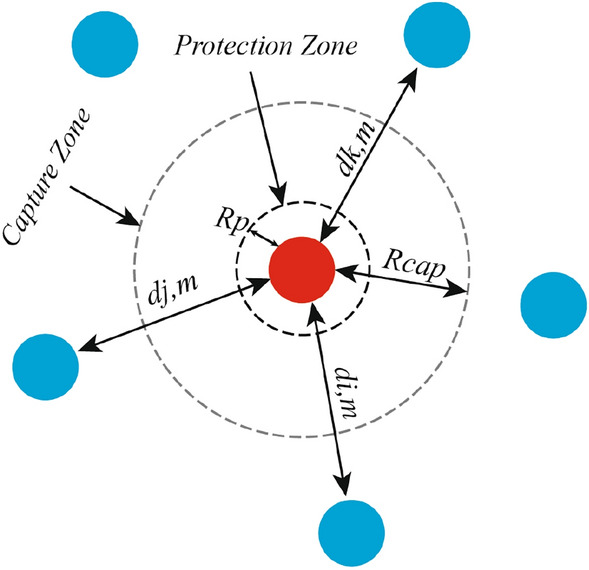
Conceptual diagram of the collaborative target roundup task.

The task requires all agents to collaboratively form a closed roundup formation within a limited number of time steps to capture the target. As shown in Fig. 1, the blue dots represent pursuing agents and the red dot denotes the escaping target. Let $d_{im}$ represent the Euclidean distance between agent *i* and target *m*. If all agents lie within a radius $R_{cap}$ of the target, and the angle between any two neighboring agents is less than a threshold $\theta _{max}$, the roundup is considered successful. A protection radius $R_{p}$ is introduced to prevent inter-agent collisions due to excessive proximity.

### Agent kinematic model

The agents’ motion is modeled using a discrete-time kinematic system:

1 $$\begin{aligned} \begin{aligned} v_{i}(t+1)&= (1 - \mu ) \cdot v_{i}(t) + a_{i}(t) \cdot \varDelta t \\ p_{i}(t+1)&= p_{i}(t) + v_{i}(t+1) \cdot \varDelta t \end{aligned} \end{aligned}$$

where $p_{i}(t)$ and ${v}_{i}(t)$ denote the position and velocity of agent *i* at time step *t*, $\varDelta t$ denotes the discrete time step, respectively, and $a_{i}(t)$ represents the acceleration vector output by the policy network of agent *i* at time step *t*^19,35^. Velocity attenuation is modeled via a damping factor $\mu \in [0,1]$, which simulates resistance in agent motion.The velocity is constrained by a maximum value $v_{max}$ to reflect physical limits.

### Escape target strategy based on APF

The escape target employs the artificial potential field (APF) method to determine its movement direction. In this approach, each agent generates a repulsive force on the target based on their relative distance, and the target moves away from the collective force exerted by the agents.

The velocity direction of the target is modeled as:

2 $$\begin{aligned} v_{m} = k_{r} \cdot \sum _{i=1}^{N} \frac{p_{m} - p_{i}}{\left\| p_{m} - p_{i} \right\| ^{2}} \end{aligned}$$

Here, $p_{m}$ and $p_i$ denote the positions of the target and agent *i*, respectively, and $k_{r}$ is the repulsive coefficient controlling the target’s response to nearby agents. The repulsive behavior allows the target to move away from the collective force field, making the scenario adversarial and dynamic.

### Roundup success criteria

The roundup is considered successful if the following two conditions are satisfied:

- $$\forall i,\ d_{im} < R_{cap}$$;
- The angle between any two adjacent agents is less than $\theta _{max}$.

Additionally, to prevent collisions caused by excessive agent proximity, the protection zone is implemented: if the inter-agent distance $d_{ij} < R_{p}$, a collision penalty is applied. These constraints are encoded into the reward function, guiding the agents to complete the task efficiently and safely.

## Multi-agent roundup algorithm design

The roundup task can be formulated as a partially observable Markov game (POMG)^22^, represented by a 6-tuple:

3 $$\begin{aligned} G = \langle \mathcal {S}, \{\mathcal {A}_i\}, P, \{r_i\}, \{\mathcal {O}_i\}, \gamma \rangle \end{aligned}$$

where:

- $\mathcal {S}$: the global state space of the environment;
- $\mathcal {A}_i$: the action space of agent *i*;
- *P*: the state transition probability function;
- $r_i$: the immediate reward received by agent *i*;
- $\mathcal {O}_i$: the local observation space of agent *i*;
- $\gamma$: the discount factor for cumulative reward.

Each agent follows a decentralized policy represented as a probability distribution over actions conditioned on its observation:

4 $$\begin{aligned} \pi _i(a_i \mid o_i): \mathcal {O}_i \rightarrow \mathcal {A}_i \end{aligned}$$

The objective of each agent is to maximize its expected long-term cumulative reward:

5 $$\begin{aligned} \max _{\pi } \mathbb {E}_{\pi } \left[ \sum _{t=0}^{\infty } \gamma ^t r_i^t \right] \end{aligned}$$

### Observation modeling and graph construction

**Fig. 2:**
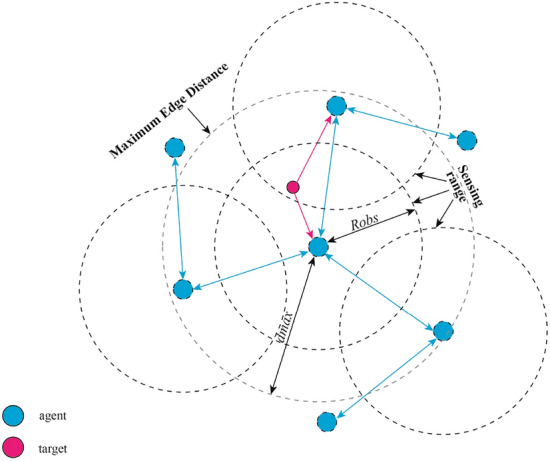
Illustration of multi-agent local sensing areas.

In this paper, each agent operates under partial observability and can only access local information within a limited sensing radius $R$, as illustrated in Fig. 2.

The sensing range defines the perceptual field of each agent, bounded by a fixed radius $R_{\textrm{obs}}$. Within this area, the agent is able to perceive the relative positions and relative velocities of neighboring entities (i.e., other agents or the target). For entities outside the sensing area, their corresponding feature entries are zero-padded, maintained to keep a fixed input dimension.

A distance threshold $d_{\text {max}}$, referred to as the maximum edge distance, is used to determine edge connectivity: an edge is formed between two entities if their Euclidean distance is less than $d_{\text {max}}$.

Based on this mechanism, we construct a dynamic graph $\mathcal {G}_t = (\mathcal {V}, \mathcal {E}_t)$, where the node set $\mathcal {V}$ includes all agents and the evading target. The edge set $\mathcal {E}_t$ is generated dynamically at each time step to reflect current adjacency relationships defined by local spatial proximity.

In our experiments, two types of observation structures are used for each agent:

- **Local observation**:
  $$o^{\text {loc}}_i = \left[ p_i,\ v_i,\ p_i^{\text {goal}},\ v_i^{\text {goal}} \right]$$
  This includes the agent’s own position and velocity, as well as the relative position and velocity of the target. It is used as the input for graph-based models under decentralized settings(e.g., GPG^17^, GAT-MARL^32^).
- **Global observation**:
  $$o^{\text {glob}}_i = \left[ p_i,\ v_i,\ p_i^{\text {goal}},\ p_{\text {other}},\ v_{\text {other}} \right]$$
  This includes additional absolute state information of all other entities, serving as input for centralized baseline algorithms including SAC^10^, MAPPO^40^, MADDPG^19^, and MATD3^1^.

#### Agent state feature

Each agent constructs a fixed-size state feature matrix that encodes the relative information from all entities in the environment, including both teammates and the target. Specifically, for each entity $j \in \{1, ..., m\}$, the corresponding feature vector is defined as:

6 $$\begin{aligned} x_{ij} = \left[ \varDelta p_{ij},\ \varDelta v_{ij},\ \text {type}_j \right] \end{aligned}$$

where $\varDelta p_{ij}$ and $\varDelta v_{ij}$ denote the relative position and velocity of entity $j$ with respect to agent $i$, and $\text {type}_j \in \{0,1\}$ indicates the entity type (0 for pursuer, 1 for target). For entities outside the sensing range of agent $i$, the corresponding feature vector $x_{ij}$ is padded with zeros.

As a result, the final input matrix for agent $i$ has a fixed shape of $\mathbb {R}^{m \times c}$, where $m$ is the total number of entities in the environment, and $c$ is the feature dimension of each entity.

### Reward function design

The reward function in this work is task-informed^38^ and is designed to reflect the core behavioral requirements of the roundup problem rather than to serve as a generic reward template for all multi-agent tasks. Specifically, the reward combines four components that correspond to complementary objectives: angular coverage encourages agents to form an enclosing geometry around the target, proximity guidance promotes efficient approach while discouraging excessive crowding near the target, roundup uniformity improves the spatial balance of the formation, and collision avoidance penalizes unsafe inter-agent spacing. This structured shaping is introduced to improve learning efficiency and stabilize coordination under partial observability, although its transferability to substantially different cooperative tasks remains a limitation to be explored in future work.

The total reward for agent *i* is defined as:

7 $$\begin{aligned} r_i = r_{\text {angle}} + r_{\text {guide}} + r_{\text {uniform}} + r_{\text {collision}} \end{aligned}$$

#### Angular coverage reward

This component promotes spatial coverage by maximizing the angular separation between agents with respect to the target:

8 $$\begin{aligned} r_{\text {angle}} = k_1 \cdot \frac{a_{ij} + a_{ik}}{4\pi } \cdot e^{-w_1 \cdot \sigma _{\theta }} \end{aligned}$$

where $a_{ij}, a_{ik}$ are the roundup angles formed between agent *i* and its adjacent neighbors, $\sigma _{\theta }$ is the standard deviation of the angles between agents and the target, and $k_1$, $w_1$ are positive constants controlling the reward scale and sensitivity.

#### Proximity guidance reward

To encourage timely convergence to the target while avoiding excessive proximity that may cause collisions or destabilize coordination, we define a piecewise reward based on the distance *d* between agent *i* and the target.

9 $$\begin{aligned} r_{\text {guide}} = {\left\{ \begin{array}{ll} \rho \ + \lambda \ \cdot \frac{R_{\text {p}} - d}{R_{\text {p}}}, & d \le R_{\text {p}} \\ - k_2 \cdot e^{w_2 \cdot d}, & d> R_{\text {p}} \end{array}\right. } \end{aligned}$$

where $R_{\text {p}}$ is the protection radius, $\rho$ is the base penalty value when the agent is closest to the target, and $\lambda$ controls the penalty variation rate within the capture radius. $k_2$, $w_2$ are tunable hyperparameters.

#### Roundup uniformity reward

To promote even distribution of agents around the target and prevent local clustering, a uniformity term is defined as:

10 $$\begin{aligned} r_{\text {uniform}} = k_3 \cdot e^{-w_3 \cdot \sigma _d} \end{aligned}$$

where $\sigma _d$ denotes the standard deviation of the distances from all agents to the target, and $k_3$, $w_3$ are positive coefficients.

#### Collision penalty

To minimize the likelihood of inter-agent collisions, we apply an exponentially decaying penalty:

11 $$\begin{aligned} r_{\text {collision}} = -k_4 \cdot e^{-w_4 \cdot d_{\min }} \end{aligned}$$

where $d_{\min }$ denotes the minimum pairwise distance between agent *i* and other agents. Smaller distances incur larger penalties, promoting safe spacing. The coefficients $k_4$ and $w_4$ are tunable parameters.

### Model architecture design: TransMARL

**Fig. 3:**
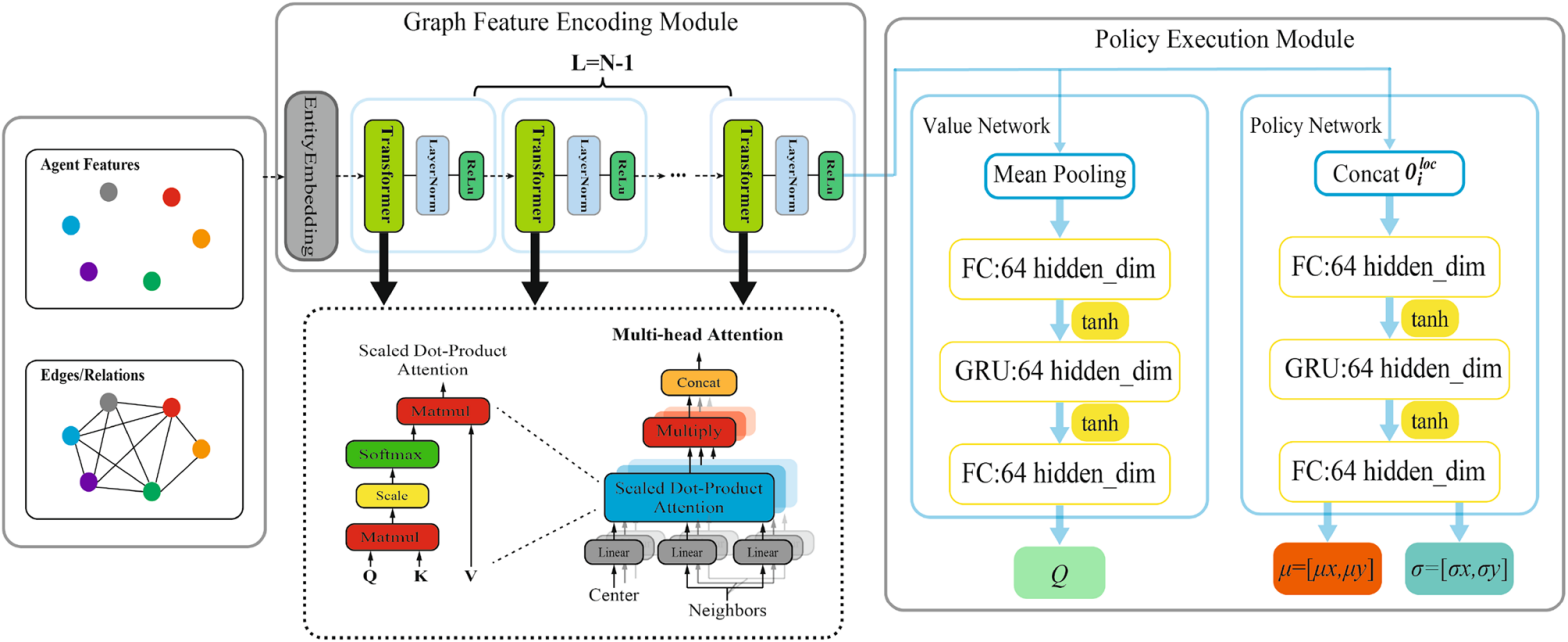
Overall architecture of TransMARL. The framework consists of the graph feature encoding module (GFEM) and the policy execution module. The GFEM encodes agent features and dynamic interaction graphs through an entity embedding layer and multiple dynamic Transformer layers, where the number of layers is adaptively set as $L=N-1$. The policy execution module contains a value network and a policy network: the value network aggregates encoded features via mean pooling to guide value estimation, while the policy network concatenates local observations with encoded features to generate action distributions.

To enable efficient feature fusion and interaction modeling in observation-constrained environments, the proposed TransMARL framework employs a hierarchical Transformer-based architecture built upon a dynamic interaction graph. The overall structure, illustrated in Fig. 3, consists of two core modules:the graph feature encoding module(GFEM) and the policy execution module. GFEM comprises the entity embedding layer and the adaptive Transformer encoding layer. Specifically, the entity embedding layer encodes heterogeneous entity types into continuous representations; the adaptive Transformer encoding layer dynamically captures complex interactions among agents; and the policy execution module produces decentralized agent actions and centralized value estimations, supporting scalable and robust multi-agent decision-making in observation-constrained environments.

### Graph feature encoding module

GFEM is responsible for extracting informative representations from the dynamic interaction graph constructed based on local observations and agent relationships. This module transforms raw input features into enriched representations that enhance interaction modeling, as illustrated in Fig. 3.

GFEM consists of two key components: the entity embedding layer, which encodes entity types and features into continuous representations, and the adaptive Transformer encoding layer, which captures inter-agent dependencies through attention-based mechanisms. The details of these components are introduced in the following subsections.

#### Entity embedding layer

The entity embedding layer encodes discrete entity types $\text {type}_j \in \{0, 1\}$ (0 for pursuer, 1 for target) into fixed-dimensional continuous vectors through a trainable embedding matrix. The resulting role-aware embeddings are then concatenated with continuous features, such as relative positions and velocities, to form comprehensive node-level input representations. Jointly optimized with subsequent model layers in an end-to-end manner, these embeddings allow the network to capture task-specific role semantics effectively through type-aware embeddings, similar to the role embedding strategies used in heterogeneous graph networks^34^. This embedding strategy is lightweight, easily extendable to additional entity types, and enhances heterogeneity-aware feature encoding, facilitating efficient downstream interaction modeling.

#### Adaptive transformer layer depth

To accommodate varying team sizes, the number of transformer layers is adjusted according to the number of agents *N*. The underlying intuition is that larger teams generally require richer relational aggregation, whereas smaller teams can often be modeled effectively with shallower interaction depth. In this work, the adaptive depth rule is adopted as a practical and empirically motivated design choice rather than a theoretically optimal solution. It is intended to balance representational capacity and computational cost by reducing underfitting in larger teams while avoiding unnecessary depth in smaller ones. We therefore interpret the adaptive-depth mechanism as a task-driven design heuristic that improves scalability in the evaluated setting, while a more rigorous theoretical characterization is left for future study.

#### Multi-head attention module

Within each Transformer layer, the model uses a multi-head self-attention mechanism to compute inter-agent relations. Concretely, each layer contains *h* parallel attention heads. Given input feature matrices *Q*, *K*, and *V* (queries, keys, and values derived from agent embeddings), each attention head performs scaled dot-product attention:

12 $$\begin{aligned} \textrm{Attention}(Q, K, V) = \textrm{softmax}\left( \frac{QK^\top }{\sqrt{d_k}} \right) V, \end{aligned}$$

as defined in Eq. 12. Here, $d_k$ is the dimension of key vectors, and learned projection matrices are used to compute *Q*, *K*, and *V*. The outputs of all heads are concatenated and linearly transformed. This multi-head design allows the model to attend to different interaction patterns simultaneously, effectively capturing diverse relational features among agents. In practice, the attention is computed over the dynamically constructed graph of agents (Section "Graph feature encoding module"), so that each agent attends primarily to its nearby neighbors.

#### Agent feature normalization

After each attention and feed-forward sublayer in the Transformer, we apply layer normalization to the agent feature vectors to improve training stability. For each agent *i*, its feature vector $x_i$ is normalized as:

13 $$\begin{aligned} \text {LN}(x_i) = \frac{x_i - \mu _i}{\sqrt{\sigma _i^2 + \epsilon }} \cdot \gamma + \beta , \end{aligned}$$

where $\mu _i$ and $\sigma _i$ denote the mean and standard deviation of $x_i$, and $\gamma$, $\beta$ are learned scaling and shifting parameters. This normalization is conducted across the feature dimensions of each agent and incorporates both the agent itself and its neighbors $\mathcal {N}(i)$ in the graph. Inspired by Ba et al.^2^, layer normalization helps to mitigate internal covariate shift, accelerates convergence, and maintains consistent feature statistics across multiple Transformer layers. In our design, it ensures that each agent’s representation remains well-scaled, which is essential for effective message propagation and stability in deep architectures.

#### Dynamic adjacency matrix construction

To define the interaction graph among agents, a dynamic adjacency matrix $A$ is constructed at each time step based on the maximum edge distance $d_{\text {max}}$. Specifically, we set

14 $$\begin{aligned} A_{ij} = {\left\{ \begin{array}{ll} 1, & \text {if } d(i,j)\ \le d_{\text {max}} \\ 0, & \text {otherwise} \end{array}\right. } \end{aligned}$$

where *d*(*i*, *j*) is the Euclidean distance between agent *i* and agent *j* (Eq. 14). This creates a time-varying graph of local connectivity: agents within sensing range are considered neighbors and able to exchange information. The adjacency matrix is updated dynamically as agents move, allowing the Transformer to encode current interactions accurately. By using a distance-based graph, the model implicitly performs message passing only among nearby agents, which effectively focuses the attention mechanism on relevant local relationships and reduces irrelevant global traffic. This dynamic graph construction endows TransMARL with the ability to handle changing topologies and partial observability in a decentralized, communication-limited setting.

### Policy execution module

The policy execution module adopts the centralized training with decentralized execution (CTDE) paradigm and consists of a decentralized actor network and a centralized critic network. This design allows each agent to make decisions based solely on local information while leveraging global feedback during training.

***Policy Network*** For each agent, the policy network takes as input the concatenation of its local observation $o_i^{\text {loc}}$ (as defined in Section "Observation modeling and graph construction") and its graph-encoded feature $h_i$. This combined input is first processed by a Gated Recurrent Unit(GRU)^8^ cell to capture temporal dependencies, followed by a multilayer perceptron (MLP) that outputs the parameters $\mu _i$ and $\log \sigma _i$ of a Gaussian policy. During training, actions are sampled from $\mathcal {N}(\mu _i, \sigma _i^2)$ with added decaying exploration noise, while in evaluation, the deterministic mean $\mu _i$ is used. The log-probability of sampled actions is computed across action dimensions. All weights in the policy network are orthogonally initialized to enhance training stability and convergence.

***Value Network*** To ensure accurate and stable value estimation, the value network constructs a global state representation by aggregating encoded agent features using a permutation-invariant pooling operation. Specifically, the global state vector $s$ is computed via average pooling:

15 $$\begin{aligned} s = \frac{1}{N}\sum _{i=1}^{N}h_i, \end{aligned}$$

where $h_i$ denotes the encoded feature of agent $i$. This operation generates a fixed-dimensional global representation that remains invariant to both agent count and ordering, naturally supporting scalability and zero-shot generalization across varying team sizes.

The resulting global state vector $s$ is processed through a GRU layer to model temporal dependencies inherent in sequential tasks, followed by an MLP to estimate the state-value function $V(s)$. Orthogonal initialization is employed for all network parameters to improve training stability. During training, a single value network is shared among all agents to ensure consistency in value estimation. During decentralized execution, only policy networks are utilized, enabling each agent to operate autonomously under partial observability conditions.

### Policy optimization procedure

**Fig. 4:**
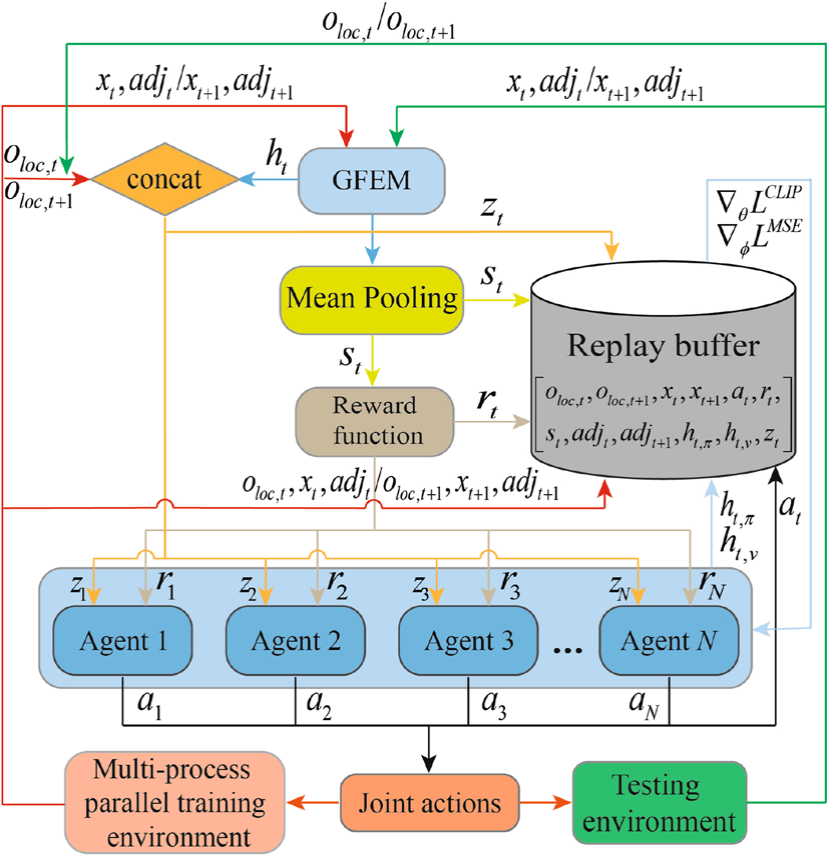
The overall policy optimization pipeline of TransMARL.

The overall policy optimization process is illustrated in Fig. 4, encompassing feature encoding, dynamic graph construction, global state aggregation, action sampling, reward computation, experience storage, and policy updates.

At each timestep $t$, the environment provides local observations $o_{loc,t}$, node features $x_t$, and adjacency matrix $A_t$. These are processed by the graph feature encoding module (GFEM) to extract higher-order interaction features $h_t$. The aggregated representation $s_t$ is then obtained via mean pooling over $h_t$, serving as the input to the value network for critic evaluation and reward computation.

The reward function calculates the immediate reward $r_t$ based on the global state $s_t$ and environmental feedback, incorporating factors such as task progress, formation quality, and collision penalties. For each agent $i$, the input to the policy network is formed by concatenating the local observation $o^i_{loc,t}$ with the corresponding encoded feature $h^i_t$, producing the multimodal feature $z^i_{t}$. This, together with the actor’s hidden state $h_{t,\pi }^{i}$, is used to generate the action $a^i_{t}$.

The joint action $a_t = \{a^i_{t}\}_{i=1}^N$ is executed in the environment, resulting in the next-step observations $o_{loc,t+1}$, node features $x_{t+1}$, and adjacency matrix $A_{t+1}$, completing the interaction for timestep $t$.

At each timestep, the following experience tuple is stored in the replay buffer:

16 $$\begin{aligned} \left\{ o_{loc,t}, x_t, A_t, z_t, a_t, r_t, s_t, v_t, h_{t,\pi }, h_{t,V}, o_{loc,t+1}, x_{t+1}, A_{t+1} \right\} \end{aligned}$$

This tuple captures all necessary information for subsequent policy and value updates, including the multimodal feature $z_t$, global state $s_t$, state value $v_t$, and the hidden states of both the actor $h_{t,\pi }$ and critic $h_{t,V}$. Once the replay buffer reaches its predefined capacity, the stored experiences are sampled to initiate policy and value updates.

#### Policy optimization

To enhance training stability and sample efficiency in policy gradient methods, this work employs generalized advantage estimation (GAE)^26^, which introduces a decay factor $\lambda \in [0,1]$ to balance the bias-variance trade-off. The advantage function is computed as:

17 $$\begin{aligned} \hat{A}_t = \delta _t + (\gamma \lambda )\delta _{t+1} + \cdots + (\gamma \lambda )^{T - t + 1}\delta _{T-1}, \end{aligned}$$

where $\delta _t = r_t + \gamma V(s_{t+1}) - V(s_t)$ represents the temporal difference (TD) error. By aggregating multi-step information with decaying weights, GAE effectively reduces variance and enhances policy update efficiency.

Based on the estimated advantages, the policy is optimized using the proximal policy optimization^27^ (PPO) algorithm. PPO employs a clipped surrogate objective to control the update step size and stabilize training:

18 $$\begin{aligned} L^{\text {clip}}(\theta ) = \mathbb {E}_t \left[ \min \left( r_t(\theta ) \hat{A}_t, \text {clip}(r_t(\theta ), 1 - \epsilon , 1 + \epsilon ) \hat{A}_t \right) \right] , \end{aligned}$$

where $r_t(\theta ) = \frac{\pi _\theta (a_t|s_t)}{\pi _{\theta _{\text {old}}}(a_t|s_t)}$ is the probability ratio between the new and old policies.

The total loss function combines the clipped policy loss, the value function loss, and an entropy regularization term:

19 $$\begin{aligned} L_{\text {total}} = L^{\text {clip}}(\theta ) + c_1 L^{\text {value}}(\theta ) + c_2 L^{\text {entropy}}(\theta ), \end{aligned}$$

where $L^{\text {value}}(\theta )$ represents the value function loss computed via mean squared error, $L^{\text {entropy}}(\theta )$ encourages exploration by penalizing overly deterministic policies, and $c_1$, $c_2$ are coefficients that balance the contributions of each component.

Policy parameters $\theta$ are updated via gradient descent on the total loss:

20 $$\begin{aligned} \nabla _\theta L_{\text {total}}(\theta ) = \nabla _\theta L^{\text {clip}}(\theta ) + c_1 \nabla _\theta L^{\text {value}}(\theta ) + c_2 \nabla _\theta L^{\text {entropy}}(\theta ). \end{aligned}$$

The Adam optimizer is employed to iteratively update both the policy and value networks, enhancing optimization efficiency while improving model stability and generalization.

**Table 1:**
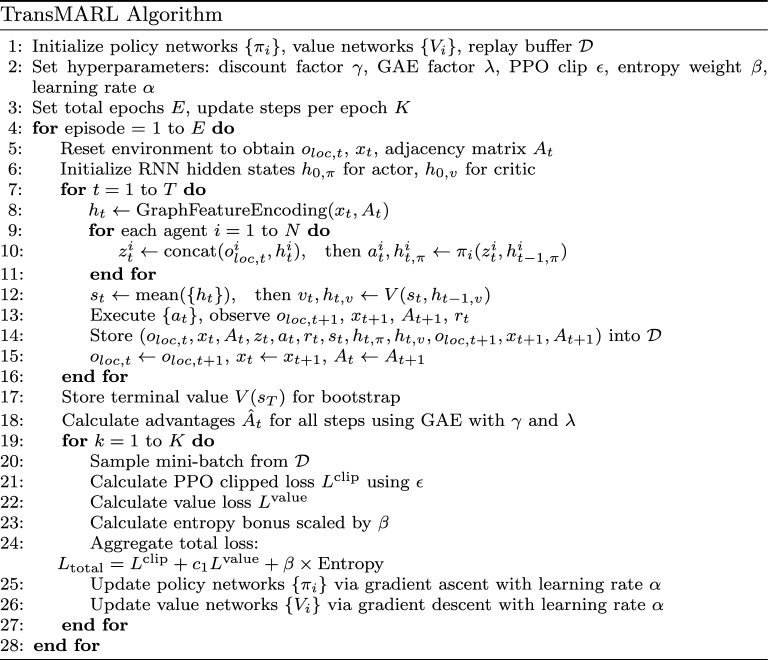
The pseudo code of TransMARL.

## Experimental design and results analysis

To validate the effectiveness and generalization capability of the proposed TransMARL method, we conduct comprehensive experiments from four perspectives: adaptive depth mechanism, local observation capability, robustness under observation constraints, and scalability. Comparisons are made against multiple baseline algorithms.

All experiments are performed in a 2D obstacle-free simulation environment where agents are initialized in an evenly spaced formation, while the relative position between the agents and the target is randomly generated, and the evading target follows the artificial potential field strategy. The detailed environment settings used in our experiments are summarized in Table 2. The corresponding parameters for the reward function design are presented in Table 3, and the key hyperparameters of TransMARL are listed in Table 4.

**Table 2:** Parameters setting for environment.

**Parameter**	**Value**
Initial velocity of agent $v_{i,0}$	0
Initial velocity of target $v_{e,0}$	0
Maximum speed of agent $v_{max}$	2
Maximum speed of target $V_{e,max}$	1.5
Maximum acceleration of agent $a_{max}$	1
Roundup radius $R_{cap}$	0.25
Protection radius $R_{p}$	0.2
Maximum edge distance $d_{max}$	1
World size	$3*3$
Maximum roundup angle $\theta _{max}$	$\pi$
Repulsive coefficient $k_{r}$	0.01
Damping factor $\mu$	0.25
Time step $\varDelta t$	1

**Table 3:** Setting of reward function parameters.

**Parameter**	**Value**
Scaling factor for angular coverage reward $k_1$	10
Base penalty within protection radius $\rho$	$-5$
Penalty variation coefficient $\lambda$	$-2$
Attraction coefficient beyond protection radius $k_2$	5
Uniformity reward coefficient $k_3$	10
Collision penalty coefficient $k_4$	5
Tunable exponential weights $w_1, w_2, w_3, w_4$	$0.1,\ 0.5,\ 0.05,\ 0.06$

**Table 4:** TransMARL hyperparameter setting.

**Parameter**	**Value**
The maximum number of episodes *E*	40000
The maximum step in each episode *T*	25
The size of experience replay buffer *M*	800
Batch process number *B*	8
Learning rate $\alpha$	0.0006
Policy entropy coefficient $\beta$	0.01
Discount factor $\gamma$	0.99
GAE parameter $\lambda$	0.95
PPO clip ratio $\epsilon$	0.2
Gradient update times *K*	15
Number of attention heads	3
Entity embedding layer dim	2
Entity embedding hidden dim	16
Transformer hidden dim	16
Optimizer	*Adam*

The maximum episode length is set to 25 steps, and all reported metrics are averaged over 100 independent trials. The evaluation metrics include:

- Success Rate ($S\%$): The proportion of episodes where the agents successfully capture the target within the step limit.
- Average Reward(*R*): The mean cumulative reward across all agents per episode.
- Collision Count (*C*): The total number of collision events caused by agents being too close during an episode.
- Average Capture Time (*T*): The average ratio of steps taken to capture the target relative to the maximum steps, with a value of 1 assigned for episodes where capture fails. A lower value indicates higher efficiency.
- Capture Quality Index (*Q*): Measures the uniformity of the agent roundup, defined as:

21 $$\begin{aligned} Q = 1 - \frac{\sigma _{\theta }}{\sigma _{\text {max}}} \end{aligned}$$

where $\sigma _{\theta }$ denotes the standard deviation of the angles formed between adjacent agents and the target, $\sigma _{\text {max}}$ represents the maximum standard deviation under an extremely imbalanced scenario, and $\theta _{\text {ideal}}$ is the ideal uniform angle. A higher *Q* value (closer to 1) indicates more uniform roundup.

From an engineering perspective, the proposed model remains lightweight in the current implementation. Training was conducted on a cloud server with a 16-core CPU, 120 GB RAM, and one NVIDIA GeForce RTX 4090 GPU. For deployment, the reported checkpoint size corresponds to the policy-side model (graph encoder + actor), excluding the critic network used only during training. Because the actor input dimension is fixed, the actor checkpoint remains constant at 27 KB. The graph encoder checkpoint increases with the adaptive-depth setting, from 40 KB ($N=3, L=2$) to 100 KB ($N=7, L=6$). Accordingly, the combined policy-side checkpoint size ranges from 67 KB to 127 KB across the evaluated configurations, indicating that the model remains lightweight for small-to-medium team settings. More detailed complexity analysis for larger-scale deployments will be considered in future work.

### Adaptive depth design and experimental verification

To determine appropriate Transformer network depths for different agent team sizes, we compare several fixed-depth structures in capture tasks with varying numbers of agents, analyzing their impact on collaborative modeling capacity. Specifically, for environments with 3, 5, and 7 agents, the tested Transformer layer depths are:

- $$L \in \{1,2,3,4\}$$ for 3 agents
- $$L \in \{3,4,5,6\}$$ for 5 agents
- $$L \in \{5,6,7,8\}$$ for 7 agents

**Fig. 5:**
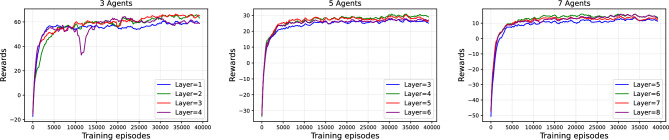
Average training reward curves of TransMARL under different Transformer depth configurations across varying team sizes ($N=3,5,7$). Each subplot illustrates the impact of layer depth on learning performance, showing that optimal depth increases with the number of agents.

Figure 5 presents the trends of average reward under each group setting. The results suggest that:

- For 3 agents, 2–3 layers achieve the best performance, while 1 layer converges faster initially but yields suboptimal final policies.
- For 5 agents, 4–5 layers perform optimally, with both shallower and deeper networks leading to unstable results.
- For 7 agents, 6–7 layers are necessary to maintain stable cooperation, as shallower settings fail to model complex interactions effectively.

These experiments reveal a consistent empirical trend: the preferred transformer depth increases with the number of agents in the evaluated settings and is often close to $L \approx N - 1$. Based on this observation, we adopt $L = N - 1$ as a simple adaptive depth rule in the remainder of this paper. We emphasize that this rule is not claimed to be theoretically optimal; rather, it serves as a practical heuristic that performed well across the current tasks. Under this interpretation, the adaptive depth setting provides a convenient way to balance expressiveness and efficiency in the present experimental setup.

#### Ablation study on the dynamic depth mechanism

To validate the practical impact of the dynamic depth mechanism in multi-agent systems, we conduct ablation experiments across environments with varying agent team sizes. Specifically, we replace the original TransMARL’s dynamic Transformer depth setting $L = N - 1$ with a fixed depth of $L = 3$, while keeping all other modules unchanged, and compare the training outcomes.

**Fig. 6:**
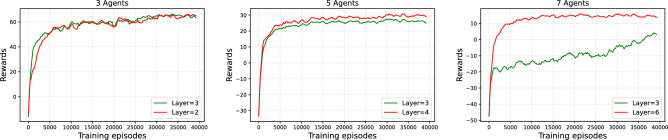
Performance comparison between fixed and dynamic Transformer layer configurations in TransMARL across different team sizes ($N=3,5,7$). The fixed-layer configuration is set to $L=3$, while the dynamic-layer configuration adjusts the depth according to team size. The results suggest that the dynamic configuration generally yields higher average rewards and more favorable convergence behavior, particularly as the number of agents increases.

Figure 6 presents the reward curve comparisons between fixed and dynamic depth strategies for teams of 3, 5, and 7 agents. The experimental observations are as follows:

- For 3 agents, both the fixed and dynamic depth strategies achieve comparable final rewards, and their training trajectories are largely consistent.
- For 5 agents, the dynamic depth strategy yields a slightly higher average reward than the fixed-depth setting, exhibiting better stability and performance ceiling.
- For 7 agents, the fixed-depth configuration struggles to model complex inter-agent interactions, with the average reward remaining in the negative region throughout training. In contrast, the dynamic depth mechanism continues to optimize the policy, ultimately achieving positive rewards, with a clear performance advantage.

These results suggest that a fixed Transformer depth can be adequate for smaller team sizes, but may become less suitable as the interaction complexity increases in the evaluated setting. By contrast, the dynamic-depth design provides a more flexible way to match the model capacity to the coordination demands of different team sizes. In particular, the advantage of the dynamic configuration becomes more apparent in larger teams, where richer interaction modeling is required. Overall, the ablation results support the view that adaptive depth is a practically useful design choice for improving coordination quality and training behavior under the tested configurations.

### Performance comparison under local observation conditions

This section evaluates the performance of TransMARL under local observation conditions by comparing it with six representative baseline algorithms. For clarity, the baselines are divided into two groups: (1) global-observation algorithms, including MAPPO, MADDPG, MATD3, and SAC, and (2) local graph-based algorithms, including GAT-MARL and GPG.

It should be noted that TransMARL, GAT-MARL, and GPG are trained under local observation, whereas MAPPO, MADDPG, MATD3, and SAC are implemented under global observation. Therefore, the results in this subsection should be interpreted as a broad empirical reference across different MARL paradigms rather than as a strictly matched comparison under identical information assumptions. In this sense, the most direct like-for-like comparison is between TransMARL and the other local-observation graph-based baselines, while the global-observation methods mainly serve as additional performance references.

**Fig. 7:**
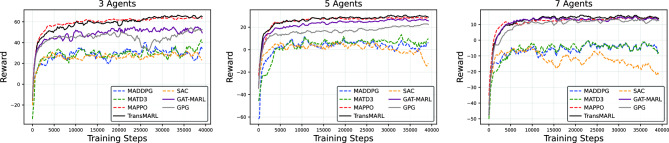
Training reward curves of different algorithms under varying team sizes ($N = 3, 5, 7$). The comparison includes both global-observation methods (MAPPO, MADDPG, MATD3, and SAC) and local graph-based methods (GAT-MARL, GPG, and TransMARL). Under the evaluated settings, TransMARL shows competitive and generally strong training performance across team sizes, particularly relative to the other local-observation graph-based baselines.

In this set of experiments, the sensing range $R_{\textrm{obs}}$ is not additionally restricted, so each agent can observe the full environment within the current simulation setup. Figure 7 shows the corresponding training reward curves for team sizes $N = 3, 5,$ and 7.

**Table 5:** Comparison of performance metrics for different algorithms under team sizes $N=3$, 5, and 7. Here, *R* denotes average reward, *C* collision count, $S\%$ success rate, *T* normalized capture time, and *Q* capture quality index.

**Algorithm**	$N=3$					$N=5$					$N=7$				
	*R*	*C*	$S\%$	*T*	*Q*	*R*	*C*	$S\%$	*T*	*Q*	*R*	*C*	$S\%$	*T*	*Q*
MADDPG	32.83	0.5	5	0.47	0.82	5.12	5.8	70	0.58	0.91	−3.13	10.4	55	0.63	0.9
MATD3	32.75	1	35	0.49	0.72	7.27	3.35	100	0.55	0.93	−2.92	10.15	95	0.65	0.87
SAC	33.91	0	0	1	N/A	5.05	2.3	0	1	N/A	−7.17	9.95	45	0.72	0.83
**MAPPO**	**62.33**	**0**	**100**	**0.24**	**0.99**	**28.27**	**1.15**	**90**	**0.38**	**0.8**	**14.03**	**23.75**	**85**	**0.39**	**0.87**
GPG	47.44	4.35	80	0.39	0.53	22.63	0.15	0	1	N/A	11.95	25.7	15	0.65	0.83
**GAT-MARL**	**53.45**	**3.8**	**95**	**0.34**	**0.6**	**27.63**	**4.15**	**95**	**0.33**	**0.82**	**13.79**	**20.3**	**85**	**0.49**	**0.85**
**TransMARL**	**65.64**	**0**	**100**	**0.15**	**0.88**	**30.04**	**0.8**	**100**	**0.17**	**0.91**	**14.47**	**23.3**	**100**	**0.22**	**0.87**

Table 5 summarizes the convergence performance of all algorithms under team sizes $N=3$, 5, and 7. The experimental findings are summarized as follows:

- For 3 agents: TransMARL achieves the highest average reward (65.64), outperforming GAT-MARL (53.45) and MAPPO (62.33). It also achieves a 100% success rate, zero collisions ($C = 0$), the shortest capture time ($T = 0.09$), and the highest capture quality ($Q = 0.91$).
- For 5 agents: TransMARL attains an average reward of 30.04, slightly higher than GAT-MARL (27.63) and MAPPO (28.27). It maintains a 100% success rate, records the shortest capture time ($T = 0.17$), achieves the lowest collision count ($C = 10.7$), and sustains a high capture quality ($Q = 0.89$).
- For 7 agents: TransMARL records an average reward of 14.47, with a consistent 100% success rate–exceeding the 85% success rate of GAT-MARL and MAPPO. Although the collision count is relatively higher ($C = 23.3$), TransMARL still achieves the shortest capture time ($T = 0.23$) and a competitive capture quality index ($Q = 0.87$).

Overall, TransMARL shows relatively strong and stable performance across the tested team sizes in the current experimental setting. The results indicate that the proposed framework can maintain high success rates and favorable capture efficiency while operating with a local-observation design. In particular, compared with the local graph-based baselines, TransMARL exhibits more consistent coordination quality and stronger training stability. At the same time, because part of the comparison involves different observation assumptions, these results should be interpreted with appropriate caution. We therefore view this experiment as additional evidence suggesting that the proposed interaction modeling strategy is beneficial in the studied setting, rather than as a definitive proof of superiority over all globally observed baselines.

### Robustness evaluation under sensing range constraints

To further examine the robustness of the proposed TransMARL method under more restrictive perception conditions, this subsection imposes explicit constraints on the sensing range $R_{\textrm{obs}}$. We evaluate capture success rates across $R_{\textrm{obs}} \in [0.3, 1.0]$, with an interval of 0.1, for TransMARL, MAPPO, and GAT-MARL under team sizes $N = \{3, 5, 7\}$. Each configuration is evaluated over 50 independent runs using different random seeds, so that the reported results reflect not only performance under restricted sensing but also the sensitivity of each method to stochastic initialization and training variation. The definition of successful capture remains the same as in the previous experiments. Here, MAPPO is included as a reference baseline under the same sensing-range evaluation protocol, although its original design is based on a global-observation training paradigm. In particular, $R_{\textrm{obs}} = 0.3$ represents the most restrictive sensing condition considered in the evaluated range and serves as an extreme case within the current experimental setting.

**Fig. 8:**
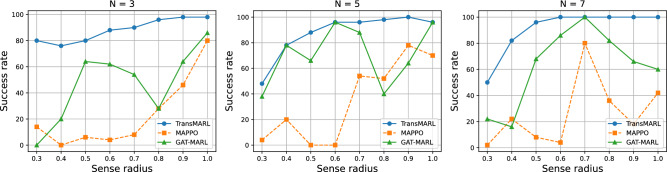
Success rates of TransMARL, MAPPO, and GAT-MARL across varying sensing radii $R_{\textrm{obs}}$ under different agent team sizes ($N=3,5,7$). The results illustrate the relative performance of the compared methods under progressively restricted local observations. Within the evaluated range, TransMARL maintains comparatively higher capture success rates than the selected baselines, particularly under low sensing radii.

As illustrated in Fig. 8, TransMARL maintains comparatively stronger performance trends under reduced sensing radii across all tested team sizes. In particular, the method degrades more gracefully than the selected baselines as local perception becomes increasingly limited, suggesting that the proposed dynamic interaction modeling is beneficial in this restricted-observation setting.

- At $R_{\textrm{obs}}=0.4$, TransMARL maintains a success rate of approximately 80%, whereas MAPPO falls below 20%, and GAT-MARL also performs substantially worse. This suggests that, within the evaluated setting, the selected baselines are more sensitive to restricted perception than TransMARL.
- As $R_{\textrm{obs}}$ increases, TransMARL’s success rate quickly approaches saturation, reaching nearly 100% when $R_{\textrm{obs}}\ge 0.6$. This trend is consistent with the view that the proposed interaction modeling becomes more effective as more local information becomes available.
- In contrast, GAT-MARL shows noticeable fluctuations, particularly with $N=5$, while MAPPO remains less competitive in this restricted-sensing evaluation. These results suggest that the selected baselines adapt less effectively than TransMARL when the available local information becomes limited.

**Fig. 9:**
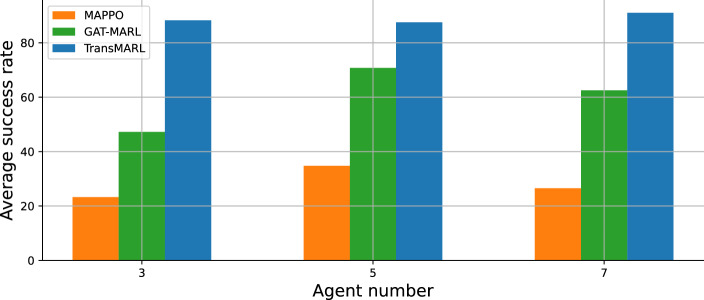
Average success rates of MAPPO, GAT-MARL, and TransMARL under different sensing radii $R_{\textrm{obs}}$ for agent team sizes of 3, 5, and 7.

To further quantify robustness under sensing restriction, we compute the average success rate for each algorithm across the full range $R_{\textrm{obs}} \in [0.3, 1.0]$, as shown in Fig. 9.

These experiments provide additional evidence that TransMARL maintains relatively strong pursuit performance under severely constrained sensing conditions within the evaluated range. As shown in Figure 9, TransMARL achieves average success rates of 88%, 90%, and 92% for agent team sizes of $N=3, 5, 7$, respectively, corresponding to an improvement of approximately 20%–40% over GAT-MARL and more than 60% over MAPPO in this sensing-range stress test. Across the evaluated team sizes, TransMARL consistently achieves higher average success rates than the selected baselines, suggesting stronger robustness to perception restriction in the current task setting.

**Fig. 10:**
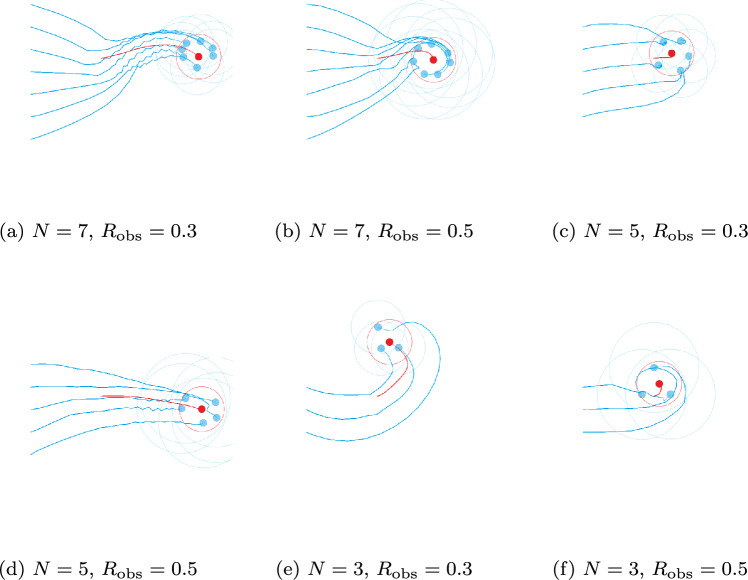
Representative pursuit and roundup trajectories of TransMARL under different sensing radii ($R_{\textrm{obs}}=0.3$ and 0.5) and team sizes ($N=3,5,7$).

To further provide qualitative insights, Fig. 10 visualizes representative pursuit and roundup trajectories achieved by TransMARL under low sensing range conditions. Specifically, the visualizations correspond to $R_{\textrm{obs}}=0.3$ and $R_{\textrm{obs}}=0.5$ across team sizes of $N=3, 5, 7$. Since the success rate exceeds 90% when $R_{\textrm{obs}}> 0.5$, we focus on lower sensing radii where perceptual limitations are more pronounced. The results show that, even under restricted observations, the agents can still coordinate effectively and complete the roundup task in representative cases.

**Fig. 11:**
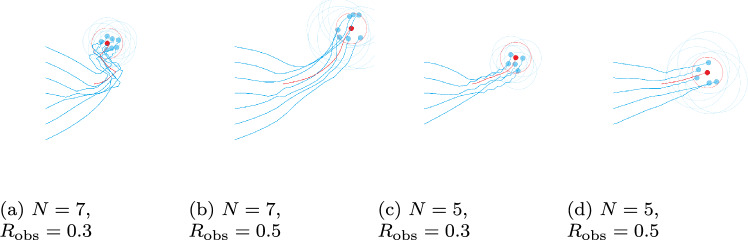
Successful pursuit and roundup trajectories of GAT-MARL under different sensing radii and team sizes.

Compared with TransMARL, the models trained using GAT-MARL exhibit partial capability to accomplish the roundup task. However, as illustrated in Figure 11, the resulting formations are less uniform, with disorganized and overly dense agent configurations that increase the risk of collisions.

**Fig. 12:**
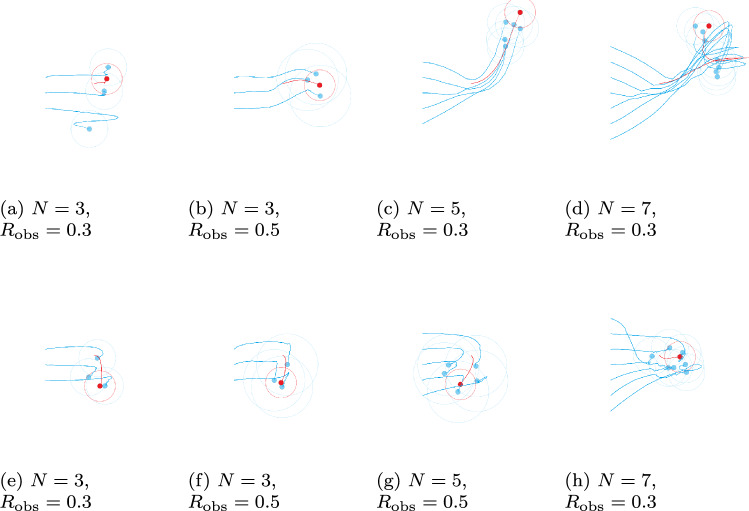
Failed pursuit and roundup trajectories of GAT-MARL (**a**–**d**) and MAPPO (**e**–**h**) under different sensing radii $R_{\textrm{obs}}$ and agent team sizes *N*.

Figure 12 presents representative failure cases of GAT-MARL and MAPPO in the roundup task. In Fig. 12(a), limited perceptual capacity hinders effective cooperation among agents, resulting in one agent losing track of the target and becoming isolated. In Fig. 12(b), although the expanded sensing radius allows agents to perceive the target and enter the roundup region, the capture condition is still not satisfied because the roundup angles remain larger than $\pi$. Similarly, in Fig. 12(c)–(h), the criteria for successful capture are unmet because not all agents reach the roundup region, and their spatial distribution remains overly dispersed. As a result, all these cases lead to unsuccessful capture attempts.

Under constrained sensing conditions, MAPPO demonstrates weaker performance in this evaluation, partly because limited local information makes cooperative policy learning more difficult in the present setting. Moreover, the limited number of steps per episode, as defined in the experimental setup, further restricts the pursuers’ ability to explore the environment and locate the evader, thereby reducing the effectiveness of coordination. In contrast, graph-based MARL algorithms generally show better adaptability in this setting because message-passing mechanisms allow agents to aggregate local observations and form a more informative shared interaction context within the available sensing range.

TransMARL shows stronger performance than GAT-MARL in this task for two main reasons. First, the dynamic-layer Transformer mechanism allows the model to adjust the depth of interaction modeling according to the number of agents. This flexibility helps maintain effective information aggregation when perception becomes more limited. Second, TransMARL employs a perception-aware dynamic graph construction strategy that updates the adjacency graph in real time based on the current sensing radius and environmental dynamics, enabling a more adaptive representation of inter-agent relationships^29^. By contrast, GAT-MARL relies on static adjacency matrices, which restrict its ability to capture changing interaction patterns^32^. These results suggest that, within the current roundup setting, TransMARL can better handle severe observation constraints than the selected baselines by improving adaptive interaction modeling under restricted local information. However, scenarios involving explicit occlusions, more complex environment structures, or broader out-of-distribution variations are beyond the scope of the present experiments and remain important directions for future work.

### Preliminary observation of cross-scale transferability

To obtain a preliminary qualitative assessment of cross-scale transferability, we directly deployed the TransMARL model trained on a 7-agent scenario ($N=7$) to new tasks with agent numbers $N = 3, 4, 5, 6$ without additional hyperparameter tuning. The observed behaviors suggest that the policy retains basic target pursuit capability across the tested team sizes, indicating a degree of transferability under this limited evaluation setting. In representative cases, successful captures were still achieved within reasonable time steps.

It should also be noted that the geometric constraints of the roundup formation (e.g., ideal angular spacing) depend on the number of agents. As a result, when the trained policy is directly transferred to new team sizes without structural adjustment, the quality of the final roundup formation declines. This observation suggests that, although the learned policy preserves a certain level of pursuit capability without fine-tuning, achieving high-quality roundup across scales may still require additional structural adaptation mechanisms, such as dynamic adjustment of angular constraints or formation-aware policy modulation.

Given that this experiment is intended only as a preliminary qualitative assessment of transferability rather than a full cross-scale generalization benchmark, we omit detailed performance curves. Accordingly, the results should be interpreted as an initial qualitative reference for scalability, while more systematic evaluation remains for future work.

## Conclusion and future directions

In this study, we proposed TransMARL, a transformer-based multi-agent reinforcement learning framework for cooperative roundup under local observation constraints. By combining dynamic interaction graph construction, adaptive-depth relational encoding, and a size-invariant policy design, the method supports decentralized coordination under partial observability. Experimental results in the evaluated 2D simulation setting show that TransMARL achieves competitive and generally strong performance across different team sizes, particularly under restricted sensing conditions.

Several limitations should be noted. The adaptive depth rule used in this work is empirically motivated and does not yet have a formal theoretical justification. In addition, all experiments were conducted in a 2D obstacle-free environment with a specific evader strategy, and the current results may therefore not directly generalize to more complex scenarios. The reward function also includes task-informed shaping terms that improve training efficiency for the roundup objective, but may introduce bias toward this specific task formulation. Moreover, some baseline comparisons involve different observation assumptions and should be interpreted with appropriate caution.

Future work will focus on providing a clearer theoretical basis for adaptive depth selection, evaluating the framework under more strictly matched comparison settings, and extending the method to more realistic environments with obstacles, heterogeneous sensing, and more complex target behaviors. Overall, the present work should be regarded as an empirical step toward scalable multi-agent coordination under observation constraints.

## Data Availability

The datasets generated and/or analysed during the current study are available from the corresponding author (Qingjia Chi, qingjia@whut.edu.cn) on reasonable request.
